# Enzyme-free ultrasensitive fluorescence detection of epithelial cell adhesion molecules based on a toehold-aided DNA recycling amplification strategy†

**DOI:** 10.1039/c8ra01362d

**Published:** 2018-04-19

**Authors:** Jishun Chen, Bing Shang, Hua Zhang, Zhengpeng Zhu, Long Chen, Hongmei Wang, Fengying Ran, Qinhua Chen, Jun Chen

**Affiliations:** Affiliated Dongfeng Hospital, Hubei University of Medicine Hubei Shiyan 442008 China cqh77@163.com

## Abstract

Epithelial cell adhesion molecules (EpCAMs) play a significant role in tumorigenesis and tumor development. EpCAMs are considered to be tumor signaling molecules for cancer diagnosis, prognosis and therapy. Herein, an enzyme-free and highly sensitive fluorescent biosensor, with a combined aptamer-based EpCAM recognition and toehold-aided DNA recycling amplification strategy, was developed for sensitive and specific fluorescence detection of EpCAMs. Due to highly specific binding between EpCAMs and corresponding aptamers, strand a, which is released from the complex of aptamer/strand a in the presence of EpCAMs which is bound to the corresponding aptamer, triggered the toehold-mediated strand displacement process. An amplified fluorescent signal was achieved by recycling strand a for ultrasensitive EpCAM detection with a detection limit as low as 0.1 ng mL^−1^, which was comparable or superior to that of reported immunoassays and biosensor strategies. In addition, high selectivity towards EpCAMs was exhibited when other proteins were selected as control proteins. Finally, this strategy was successfully used for the ultrasensitive fluorescence detection of EpCAMs in human serum samples with satisfactory results. Importantly, the present strategy may be also expanded for the detection of other targets using the corresponding aptamers.

## Abstract 2

A fluorescent biosensor with a combined aptamer-based EpCAM recognition and toehold-aided DNA recycling amplification strategy was developed.

## Graphical abstract

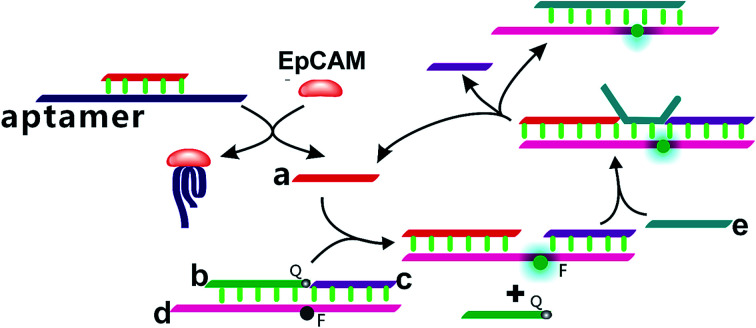

## 1. Introduction

Nowadays, cancer has become a worldwide problem which threatens public health and is the leading cause of death in China and the second leading cause of death in the United States.^1,2^ Efforts in the fight against cancer will need sustained clinical and basic research to improve the effectiveness of diagnostic techniques and screening programs, which is critical for reversing the cancer epidemic worldwide.^2^ Thus, the development of effective methods for cancer therapy has attracted increasing worldwide attention in the medical field. Among them, the early identifying and quantifying of carcinoma biomarkers could provide an easier and more effective way to monitor the progression of carcinomas, which is of great importance for early accurate diagnosis and effective therapy for cancer.^3,4^

EpCAM, a glycosylated transmembrane protein which is normally expressed in many epithelial tissues throughout the body, mediates epithelial-specific intercellular cell adhesion and is involved in cell signal transduction, proliferation, migration, differentiation and invasion.^5–7^ However, in subsequent studies, overexpression of EpCAM was also found in human colon carcinomas,^8^ breast cancer,^9,10^ pancreatic cancer,^11^ gallbladder cancer,^12^ gastric cancer^13^ and so on, but low levels or no expression in normal healthy tissues.^14,15^ Thus, the overexpression of EpCAM has been regarded as a prognostic tumor biomarker associated with a poorer prognosis in a wide variety of different carcinomas and reflects the existence and growth of tumors in the human body.^7^ Due to this differential expression of EpCAM between human cancers and normal healthy cells, EpCAM plays a significant role in tumorigenesis and tumor development and it is considered to be one of the prognostic tumor signaling molecules for cancer diagnosis, prognosis and therapy.^16,17^ Therefore, the early sensitive and reliable detection of EpCAM is of great significance for the early clinical diagnosis of tumors. Considering the significant role of EpCAM in the early diagnosis of tumors, more attention has been given to developing quantitative methods for the detection of EpCAM in the past few decades. Many diagnostic strategies relying on anti-EpCAM antibodies have been developed.^18,19^ Among these, enzyme-linked immunosorbent assays (ELISA) represent the major approach for the sensitive detection of EpCAM.^7,20,21^ However, this antibody-based method is usually labor intensive, complicated, expensive, time-consuming and even requires highly skilled personnel. To date, only a few novel strategies, including electrochemical biosensors^20^ and fluorescence biosensors,^17,22^ have been developed for the sensitive determination of EpCAM. Among them, fluorescence biosensors are particularly attractive due to their high sensitivity, easy readout, simplicity and the feasibility of quantification.^23,24^

Aptamers are single-stranded functional DNA or RNA structures that are obtained *in vitro* from large random-sequence nucleic acid libraries by the systematic evolution of ligands by exponential enrichment (SELEX) technology.^25–27^ They are capable of easily recognizing and binding specific targets including metal ions, small molecules, proteins and even whole viruses or cells.^28,29^ In comparison with antibodies, aptamers possess numerous unique advantages including design flexibility, ease of modification, easy and controllable labeling, high specificity, high purity, long-term stability and so on.^4,24^ Based on these merits, they have been attracting increasing research efforts as alternative bio-recognition elements to antibodies for biosensor design. Meanwhile, the ratio of aptamer to target is 1 : 1 in almost all technologies, resulting in low sensitivity and a high error rate. The sensitivity of these reported aptamer-based fluorescence detection systems is compromised. To overcome these limitations, signal amplification strategies, including enzyme-aided signal amplification (nicking endonucleases, exonucleases, DNAzymes, *etc.*),^23,30–33^ catalyzed hairpin assembly (CHA),^34^ molecular machines,^35,36^ the hybridization chain reaction (HCR),^37^ rolling circle amplification,^38^ nanoparticle-assisted amplification^39^ and toehold-aided DNA recycling amplification,^40,41^ have recently been developed to achieve the sensitive detection of biomolecules in the field of bio-analytical sciences. Among these signal amplification strategies, toehold-aided DNA recycling amplification has the advantages of being enzyme-free, easy to use and inexpensive, having continuous signal turnover capability and inherent modularity and being easy to scale up,^32,33^ and is especially intriguing for signal amplification. Toehold-aided DNA recycling amplification has overcome the disadvantages of the specific reaction conditions and reaction time dependent enzyme activity of enzyme-aided signal amplification,^34^ and reversed the low specificity caused by great background signals due to nonspecific CHA products in the absence of a target.^42^

Toehold-aided DNA recycling amplification is a controllable independent process based on a toehold-mediated strand displacement process, without the participation of various enzymes or nanomaterials, which does not have the disadvantages of expensive price, poor stability, complicated operation, specific reaction conditions or reaction time dependent enzyme activity.^34^ It was firstly pioneered for the construction of a tweezer-like dynamic molecular machine by Yurke *et al.*^36,40^ A toehold, a short single-strand overhanging domain of double-stranded complex to which the target sequence attaches and then compels one DNA strand in a double-stranded complex to migrate away, triggers the strand displacement process.^33,43^ Toehold-mediated strand displacement exhibited high sequence-dependence and was successfully applied to the construction of nanomachines,^36,44^ molecular self-assembly,^45^ logic gates,^46,47^ signal amplification,^40^ neural networks^48^ and so on. To meet the demands of the specificity, sensitivity and feasibility of EpCAM detection, the development of an enzyme-free signal amplification strategy is extremely urgent.

Considering the specificity of aptamer-based biosensors and the intriguing characteristics of toehold-aided DNA recycling amplification, the combination of the two strategies is promising for the specific and sensitive detection of EpCAM. Herein, we report an aptamer-based enzyme-free approach for the ultrasensitive fluorescence detection of EpCAM using a toehold-aided DNA recycling amplification strategy. The toehold-aided DNA recycling amplification strategy, with cyclic reuse of the initiation strand (strand a) for the direct fluorescence detection of EpCAM, was developed successfully for the ultrasensitive detection of EpCAM at levels as low as 1.0 ng mL^−1^, with a linear range from 2 ng mL^−1^ to 150 ng mL^−1^. Moreover, the suitability of this approach for the sensitive determination of EpCAM in real serum was also investigated, with recovery in the range of 109.2–114.8%. Therefore, the developed strategy will become a promising and reliable method for the ultrasensitive detection of EpCAM in the early clinical diagnosis of cancers and medical research.

## 2. Experimental section

### 2.1. Reagents and materials

EpCAM, bovine serum, CD86 and CD63 were purchased from Cusabio Biotech Co. Ltd. The aptamers and synthetic DNA sequences (Table 1) were all purchased from Sangon Biotechnology Co. Ltd. (Shanghai, China, www.sangon.com) and purified using HPLC. The other reagents employed were of analytical grade and used without further purification. All reagents were diluted to the required concentration with working buffer (20 mM Tris–HCl, 100 mM NaCl, 10 mM KCl and 10 mM MgCl_2_; pH 7.5) before usage. Healthy human serum was obtained from the Dongfeng General Hospital. Ultrapure water prepared with a Millipore water purification system (18.2 MΩ cm resistivity, Milli-Q Direct 8) was used in all runs.

**Table 1:** The aptamer and synthetic DNA strand sequences used in this work

Name	Sequence
EpCAM aptamer	5′-CACTACAGAGGTTGCGTCTGTCCCACGTTGTCATGGGGGGTTGGCCTG-3′
Strand a (15 nt)	5′-AGACGCAACCTCTGT-3′
Strand b (13 nt)	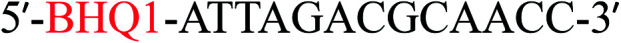
Strand c (12 nt)	5′-GATAGTACTAT-3′
Strand d (29 nt)	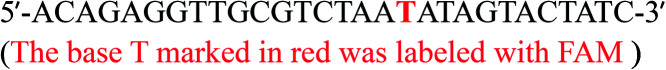
Strand e (19 nt)	5′-TACTATATTAGACGCAACC-3′

### 2.2. EpCAM sensing procedure

Prior to the experiments, the mixtures of aptamer/strand a and strand b/strand c/strand d hybridized strands were heated at 90 °C for 5 min, and then slowly cooled down to room temperature. Equimolar concentrations of strand b, strand c and strand d were mixed. Next, 100 μL EpCAM at different concentrations was incubated with 100 μL aptamer/strand a for 30 min at 37 °C. This was followed by the addition of the 100 μL mixture of strand b/strand c/strand d. Subsequently, 100 μL strand e was introduced and the solution was incubated at 37 °C. Finally, the solution was diluted to 1 mL and the fluorescence intensity of the solutions was measured.

### 2.3. Fluorescence measurements

The fluorescence detection of the mixture was carried out using a Hitachi F-4600 spectrophotometer (Hitachi Co. Ltd., Japan, www.hitachi.co.jp) equipped with a xenon lamp excitation source at room temperature. The excitation was set at 495 nm and the emission spectra were collected from 510 nm to 600 nm. The fluorescence intensity at 518 nm was used to investigate the optimal experimental conditions and evaluate the performance of the proposed sensing system. In the control experiments, the measurement process was the same as above, except for the addition of EpCAM. Unless otherwise noted, each fluorescence measurement was repeated three times, and the standard deviation was plotted as an error bar. The quantitative assay of EpCAM was realized using the fluorescence intensity. *F*_1_ and *F*_0_ are the fluorescence intensities at 518 nm in the presence and absence of EpCAM, respectively.

## 3. Results and discussion

### 3.1. Design principles

**Scheme 1:**
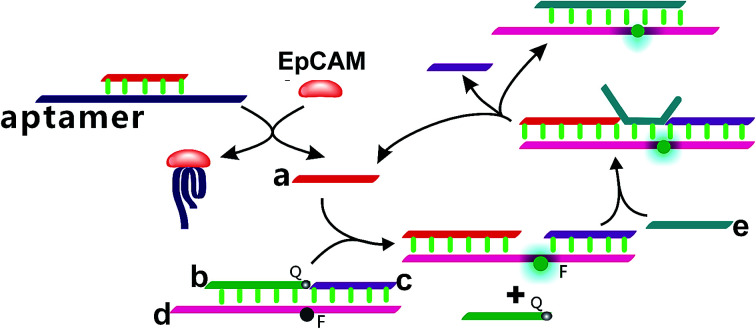
Schematic illustration of the enzyme-free fluorescence detection of EpCAM on the basis of a combined aptamer-based EpCAM recognition and toehold-aided DNA recycling amplification strategy.

In the present study, a enzyme-free fluorescence amplification strategy for sensitive EpCAM detection on the basis of a combined aptamer-based EpCAM recognition and toehold-aided DNA recycling amplification strategy was developed. As illustrated in Scheme 1, strand a, which triggers the toehold-mediated strand displacement reaction, is firstly hybridized with the EpCAM aptamer sequence to form an aptamer/strand a duplex, preventing the occurrence of a strand displacement reaction in the absence of EpCAM. Strand b, strand c and strand d are hybridized to form a b–c–d duplex with weak fluorescent emission, due to the quencher-contained strand b quenching the fluorescence of the fluorescence reporter of strand d. In the presence of EpCAM, strand a can be dissociated from the aptamer/strand a duplex due to the highly specific affinity between EpCAM and the corresponding aptamer. The liberated strand a further hybridizes with the toehold domain of the b–c–d duplex and triggers the toehold-aided strand displacement reaction, leading to the release of strand b to form an a–c–d duplex, which increases the fluorescent signal. Upon addition of strand e, it hybridizes with the toehold domain of the above formed a–c–d duplex and displaces strand a and strand c. The liberated strand a hybridizes again with the toehold domain of the b–c–d duplex and triggers a toehold-aided DNA recycling amplification, leading to significantly amplified fluorescence emission for the ultrasensitive detection of EpCAM.

### 3.2. Feasibility analysis of the developed method for EpCAM detection

**Fig. 1:**
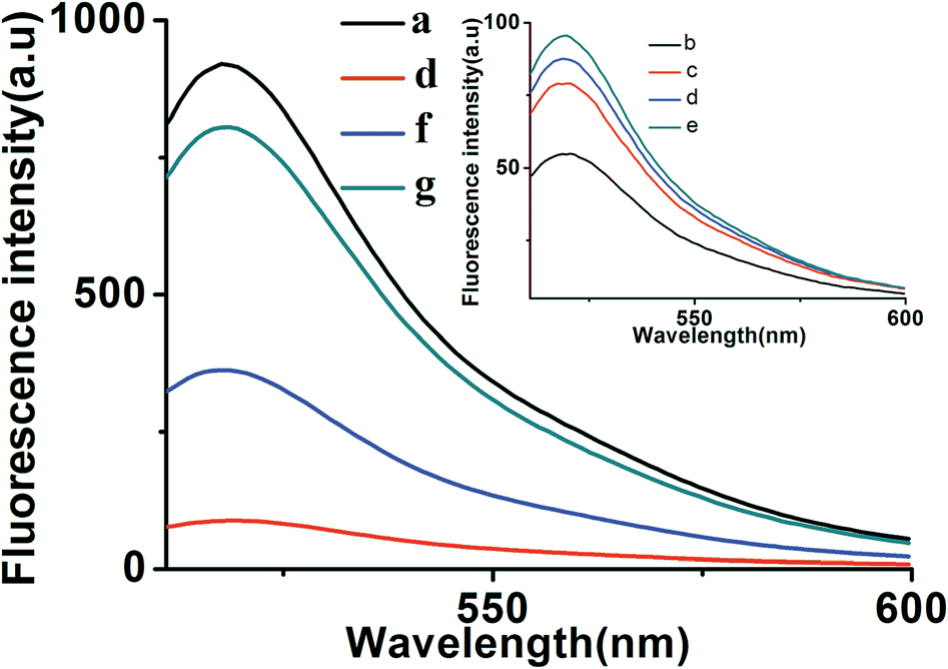
Typical fluorescent emission spectra of different mixtures. From curve a to f: (a) strand d (100 nM); (b) strand b + strand c + strand d (100 nM); (c) strand b + strand c + strand d + strand e (100 nM); (d) aptamer/strand a + strand b + strand c + strand d + strand e (aptamer/strand a: 30 nM; strand b, c, d and e: 100 nM); (e) aptamer/strand a + strand b + strand c + strand d + EpCAM (aptamer/strand a: 30 nM; strand b, c and d: 100 nM); (f) aptamer/strand a + strand b + strand c + strand d + strand e + EpCAM (aptamer/strand a: 30 nM; strand b, c, d and e: 100 nM; EpCAM: 50 ng mL^−1^); (g) strand a + strand b + strand c + strand d + strand e (strand a: 30 nM; strand b, c, d and e: 100 nM).

**Fig. 2:**
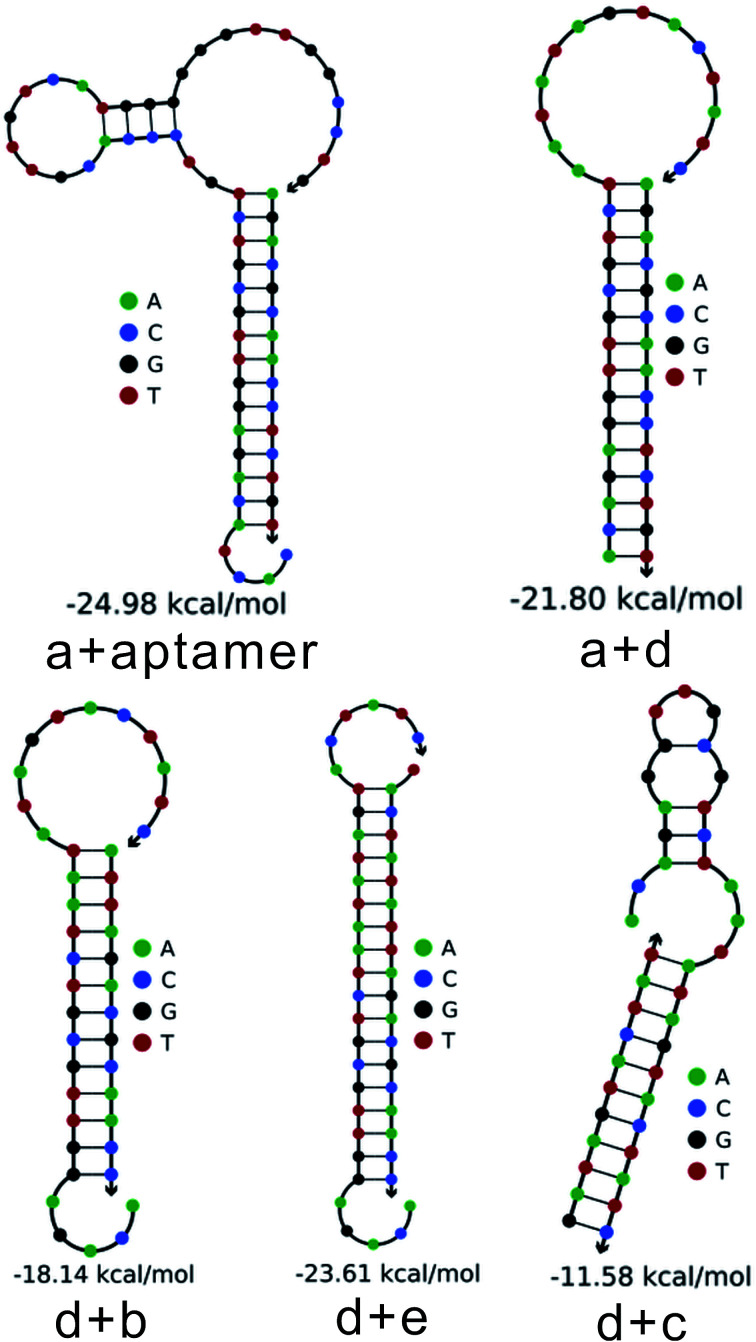
The secondary structures and the free energy of the formation of the corresponding duplexes at 37 °C (the incubation temperature) were analyzed using online software (http://www.nupack.org/); strand a + aptamer, strand a + strand d, strand d + strand b, strand d + strand e and strand d + strand c.

To further verify the feasibility of the toehold-aided DNA recycling fluorescent signal amplification strategy, fluorescence measurements were performed to record the fluorescence emission spectra of different mixtures. As shown in Fig. 1, when compared with the highly fluorescent signal of DNA strand d (curve a), an extremely weak fluorescent signal for the b–c–d duplex was obtained, which was attributed to the quencher-contained strand b quenching the fluorescence of the fluorescence reporter of strand d (curve b *vs.* curve a). On the addition of strand e, a very slightly increased fluorescent signal was observed (curve c *vs.* curve b), indicating the partial dissociation of strand b from the b–c–d duplex in the presence of strand e. The reason for this may be that, although the binding capacity of strand e to strand d is greater than that of strand b and strand c to strand d, there were no unpaired bases to hybridize with strand e when strand d was first hybridized with strand b and strand c, resulting in the slow reaction of strand e displacing strand b and strand c over a short period of time. Similarly, the incubation of aptamer/strand a, b, c, d and e showed negligible fluorescence intensity changes compared with that of strand b, c, d and e (curve d *vs.* curve c). On the addition of strand e, a significant enhancement in fluorescence intensity was further exhibited in the presence of EpCAM (curve f *vs.* curve e). This apparent signal enhancement further indicated the successful release of strand a from the a–c–d duplex and subsequent toehold-aided DNA recycling amplification in the presence of strand e. A significant enhancement in fluorescence intensity was also observed in curve g, indicating the occurrence of the toehold-mediated strand displacement reaction in the presence of strand a. The gel electrophoresis results also confirmed the above results (Fig. S1†). As we all know, Gibbs free energy can also reflect the stability of a DNA duplex. A smaller Gibbs free energy value indicates better stability for the hybridization of two complementary strands. The occurrence of toehold-aided strand displacement was first based on the strands with smaller Gibbs free energy displacing the strands with larger Gibbs free energy in the presence of unpaired bases in complementary strands.^43^ Therefore, the Gibbs free energy of the formation of different duplexes was also analyzed using online software (http://www.nupack.org/). Fig. 2 shows the secondary structures and the free energy of the formation of the corresponding duplexes: strand a/aptamer, strand a + strand d, strand d + strand b, strand d + strand e and strand d + strand c, which further verifies the aforementioned results.

### 3.3. Optimization of reaction conditions

**Fig. 3:**
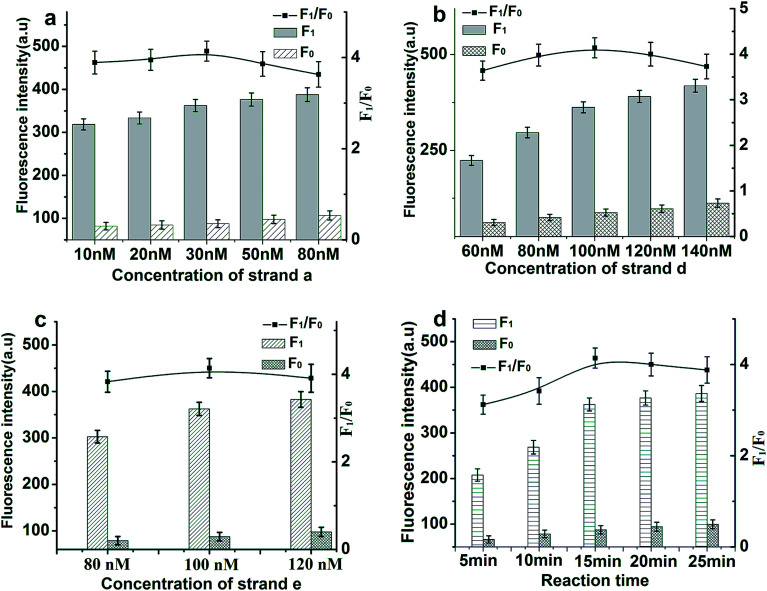
Effects of reaction conditions on the fluorescent signals and *F*_1_/*F*_0_ values of the proposed method. (a) Concentration of strand a (strand b, c, d and e: 100 nM; EpCAM: 50 ng mL^−1^). (b) Concentration of strand d (aptamer/strand a: 30 nM; strand b, c, d and e: 100 nM; EpCAM: 50 ng mL^−1^). (c) Concentration of strand e (aptamer/strand a: 30 nM; strand b, c, and d: 100 nM; EpCAM: 50 ng mL^−1^). (d) The reaction time (aptamer/strand a: 30 nM; strand b, c, d and e: 100 nM; EpCAM: 50 ng mL^−1^).

In order to achieve optimal sensing performance using the proposed toehold-aided DNA recycling amplification strategy for EpCAM detection, several reaction conditions such as the concentration of aptamer/strand a, the concentration of strand d, the concentration of strand e and the reaction time were optimized. The fluorescence intensity and the value of *F*_1_/*F*_0_ were selected to evaluate the effects of the aforementioned reaction conditions on the sensing performance of the developed method, where *F*_1_ and *F*_0_ were the fluorescence intensities of the solutions in the presence and absence of EpCAM, respectively. As depicted in Fig. 3(a), the value of *F*_1_/*F*_0_ increased gradually along with the increasing concentration of aptamer/strand a in the range from 10 nM to 30 nM and a gradual decrease appeared thereafter because of an accelerated increase in background fluorescent signals. Thus, an EpCAM aptamer/strand a concentration of 30 nM was confirmed as the optimized concentration.

The ratio of 1 : 1 : 1 for strand b (labeled with black hole quencher (BHQ1) at the 5′ end) : strand c : strand d (labeled with 6-carboxyfluorescein (FAM) at the base T, marked in red) was selected to ensure the weak fluorescent emission of the b–c–d duplex, due to the quencher-contained strand b quenching the fluorescence of the fluorescence reporter of strand d. The concentration of the b–c–d duplex and strand e are of great importance for the efficiency of toehold-aided DNA recycling amplification. Firstly, low concentrations of the b–c–d duplex and strand e may limit the fluorescent signal amplification efficiency, because a great deal of the initiation strand (strand a) or product sequences released in the reaction need to hybridize with adequate quantities of strand e or the b–c–d duplex in order to trigger toehold-aided DNA recycling amplification.^23^ Secondly, high concentrations of the b–c–d duplex and strand e may give rise to an increase in background signal because of nonspecific amplification. Therefore, the concentrations of the b–c–d duplex and strand e were also investigated. As shown in Fig. 3(b) and (c), fluorescence intensity increased gradually along with increasing concentration of the b–c–d duplex in the range from 60 nM to 140 nM and increasing concentration of strand e in the range from 80 nM to 120 nM in the presence or absence of EpCAM (50 ng mL^−1^). The *F*_1_/*F*_0_ value reached a maximum when the concentrations of the b–c-d duplex and strand e are both at 100 nM. Therefore, a concentration of 100 nM was selected as the optimized concentration of both the b–c–d duplex and strand e.

The reaction time is another important reaction condition affecting fluorescence intensity. The plots depicted in Fig. 3(d) represent changes in fluorescence intensity and *F*_1_/*F*_0_ values along with reaction time varying from 5 min to 25 min at time intervals of 5 min. The *F*_1_/*F*_0_ value reached a maximum when the reaction time was 15 min and then decreased gradually because of an accelerated increase in background fluorescent signal. Thus, 15 min was confirmed as the optimized reaction time.

### 3.4. Sensitivity for EpCAM detection

**Fig. 4:**
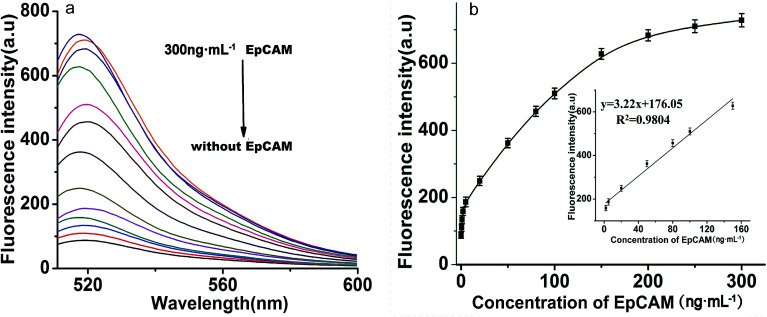
(a) Typical fluorescence emission spectra of the proposed DNA recycling amplification strategy for different concentrations of EpCAM. From the bottom to the top: 0, 0.5, 1, 2, 5, 20, 50, 80, 100, 150, 200, 250 and 300 ng mL^−1^. (b) The fluorescence intensity *vs.* the concentration of EpCAM, from 0 to 300 ng mL^−1^, at an emission wavelength of 518 nm. Inset: the linear relationship between fluorescence intensity and EpCAM concentration in the range from 2 ng mL^−1^ to 150 ng mL^−1^. Error bars: SD, *n* = 3.

Under optimized reaction conditions, the sensitivity of the proposed toehold-aided DNA recycling amplification strategy for EpCAM detection was evaluated at different concentrations. As shown in Fig. 4(a), fluorescence intensity gradually increased along with the concentration of EpCAM, from 0 to 300 ng mL^−1^. By plotting the curve of fluorescence emission intensity *vs.* concentration of EpCAM at an emission wavelength of 518 nm (shown in Fig. 4(b)), a good linear relationship between the fluorescence intensity and the concentration of EpCAM was obtained, in the range from 2 ng mL^−1^ to 150 ng mL^−1^, with a regression coefficient (*r*^2^) of 0.9804 and a detection limit of 0.1 ng mL^−1^ (obtained according to the 3*σ* rule). This low detection limit which provided excellent sensitivity was comparable to the reported immunoassays and electrochemical microfluidic immunosensor,^20^ and higher than that of the reported fluorescence biosensor for EpCAM detection.^17^ The excellent sensitivity and broad linear range indicated that this toehold-aided DNA recycling amplification strategy was satisfactory for the ultrasensitive fluorescence detection of EpCAM.

### 3.5. Specificity for EpCAM detection

**Fig. 5:**
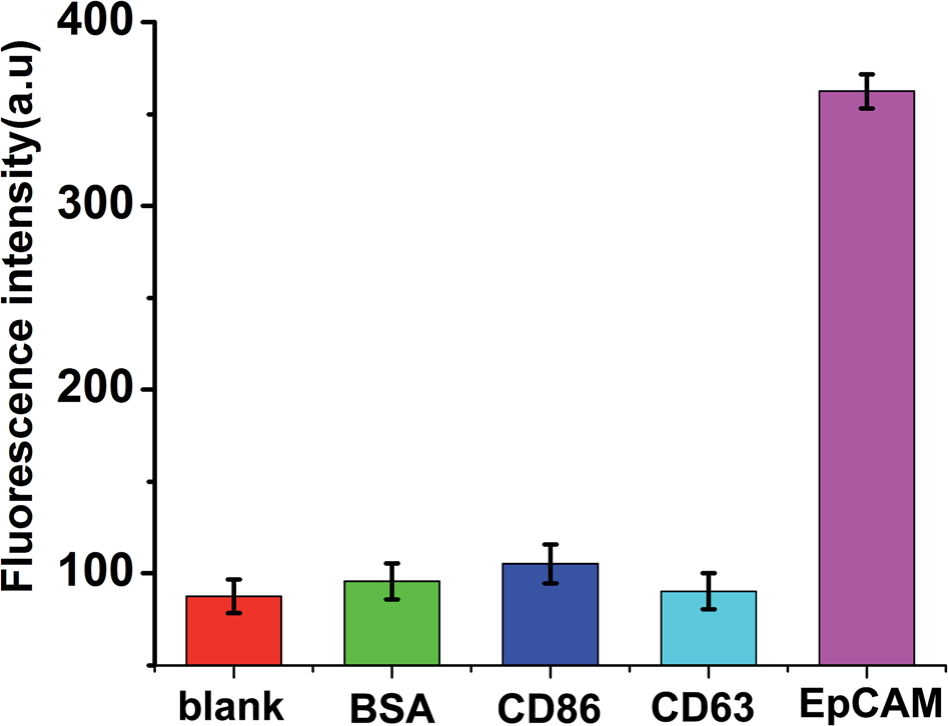
Selectivity investigation of the proposed method for the detection of EpCAM (50 ng mL^−1^), BSA (100 ng mL^−1^), CD86 (100 ng mL^−1^), CD63 (100 ng mL^−1^). Error bars: SD, *n* = 3.

Due to high recognition and specific affinity between the aptamers and the targets, the aptamer-based biosensors exhibit significant specificity. In the present work, three different relevant proteins, including BSA, CD86 and CD63, with a concentration of 100 ng mL^−1^ at 2-fold higher than that of EpCAM, were spiked respectively. The measurements were performed under the same conditions to validate the specificity of the proposed method for EpCAM detection. As shown in Fig. 5, in the presence of other control proteins (100 ng mL^−1^), slight fluorescence changes were observed in the absence of EpCAM, while a significant enhancement of fluorescence emerged in the presence of EpCAM (50 ng mL^−1^), which indicated the excellent specificity of the proposed strategy for the detection of EpCAM.

### 3.6. Determination of EpCAM in real samples

**Fig. 6:**
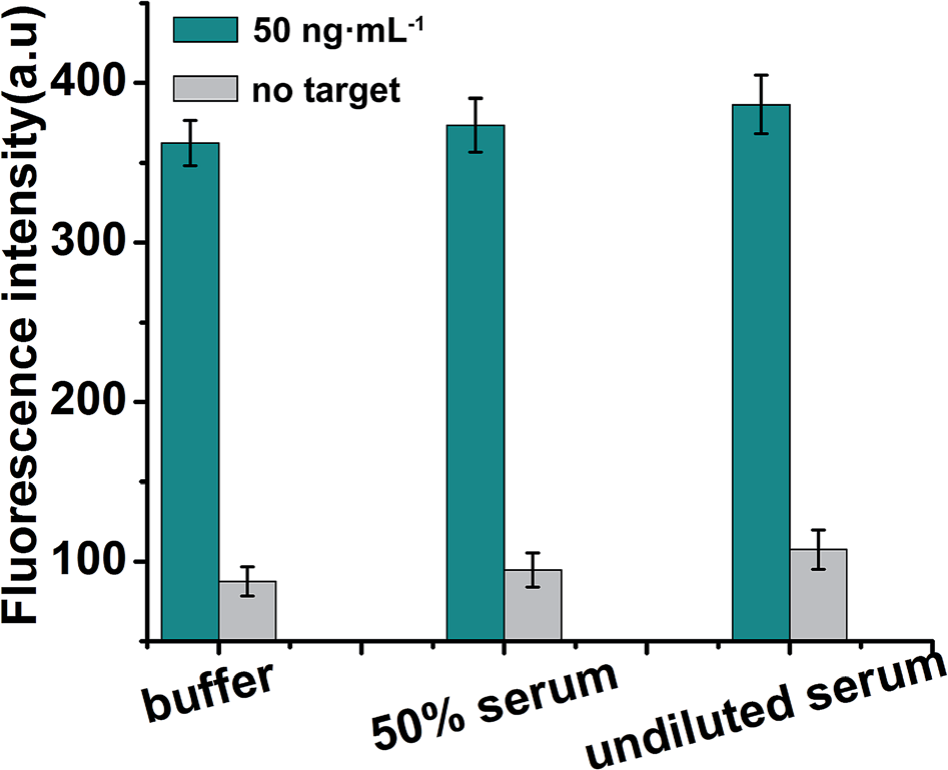
Detection of EpCAM in spiked EpCAM (50 ng mL^−1^) in buffer, 50% serum and undiluted serum. Error bars: SD, *n* = 3.

To further verify the potential applicability of the present strategy, the detection of EpCAM in biological samples by spiking human serum and 50% serum (diluted with buffer) (human serum obtained from Dongfeng General Hospital) with various concentrations of EpCAM was performed according to the EpCAM sensing procedure. As shown in Fig. 6, a significant increase in fluorescence in the presence of 50 ng mL^−1^ EpCAM in undiluted serum was observed when compared with the blank test (in the absence of EpCAM), while a negligible change in fluorescence intensity in serum was observed when compared with 50% serum or buffer (50 ng mL^−1^ EpCAM), which indicated that the detection of EpCAM in serum is free of matrix interference. The recoveries for the various concentrations of spiked EpCAM in human serum were in the range of 109.2–114.8%, with the relative standard deviations (RSDs) of 15.6%, 18.2% and 17.5% at 5, 50 and 100 ng mL^−1^ of EpCAM, respectively, indicating the acceptable precision and reproducibility of the present approach for detecting EpCAM in real samples (*n* = 3) (Table 2).

**Table 2:** Recovery of EpCAM spiked in human serum samples

Sample	EpCAM added/ng	EpCAM found/ng	Recovery (100%)	RSD (%)
1	0	0	—	—
2	5.0	5.6^a^	112.0	15.6
3	50.0	54.6^a^	109.2	18.2
4	100.0	114.8^a^	114.8	17.5

a The mean values of the three measurements.

## 4. Conclusions

In summary, a fluorescence biosensor for ultrasensitive EpCAM detection was firstly constructed by combining toehold-aided DNA recycling amplification with aptamer-based target recognition, without the participation of enzymes. Due to a significant fluorescent amplification signal in response to EpCAM and aptamer-based EpCAM recognition, the sensitive and specific detection of EpCAM was achieved, with a detection limit as low as 0.1 ng mL^−1^. In addition, this approach has been successfully applied in the specific and sensitive detection of EpCAM in real samples, indicating that it will become a reliable method for EpCAM detection in the early clinical diagnosis of cancers. Moreover, the present enzyme-free ultrasensitive fluorescence biosensing strategy may be also a promising strategy for the direct detection of other biomarkers by selecting the corresponding aptamers in early clinical diagnosis.

## Conflicts of interest

There are no conflicts to declare.

## Supplementary Material

RA-008-C8RA01362D-s001
