# Supplementary material for: Enzyme-free ultrasensitive fluorescence detection of epithelial cell adhesion molecules based on a toehold-aided DNA recycling amplification strategy
Source: RSC Adv. 2018 Apr 19;8(27):14798–805. doi: 10.1039/c8ra01362d (PMC9079946; doi:10.1039/c8ra01362d)
Supplement: RA-008-C8RA01362D-s001 [file RA-008-C8RA01362D-s001.pdf]

Electronic Supplementary Material (ESI) for RSC Advances.
This journal is © The Royal Society of Chemistry 2018

Electronic supplementary information for RSC Advances

Enzyme-free ultrasensitive fluorescence detection of epithelial cell adhesion molecule based on toehold-aided DNA recycling amplification strategy

Jishun Chen [#], Bing Shang [#], Hua Zhang, Zhengpeng Zhu, Long Chen, Hongmei Wang, Fengying Ran, Qinhua Chen, Jun Chen**

Affiliated Dongfeng Hospital, Hubei University of Medicine, Hubei Shiyan 442008, China

Jishun Chen and Bing Shang are all the first authors.

Qinhua Chen and Jun Chen are all the corresponding authors. E-mail address: cqh77@163.com (Q. Chen)

Native polyacrylamide gel electrophoresis

The different DNA mixed solutions were analyzed in 12% polyacrylamide gel. Electrophoretic analysis was carried out in 1 × TBE by DYCZ-25D electrophoretic apparatus at a constant voltage of 75 V for 100 min. The gel was stained by GelRed.

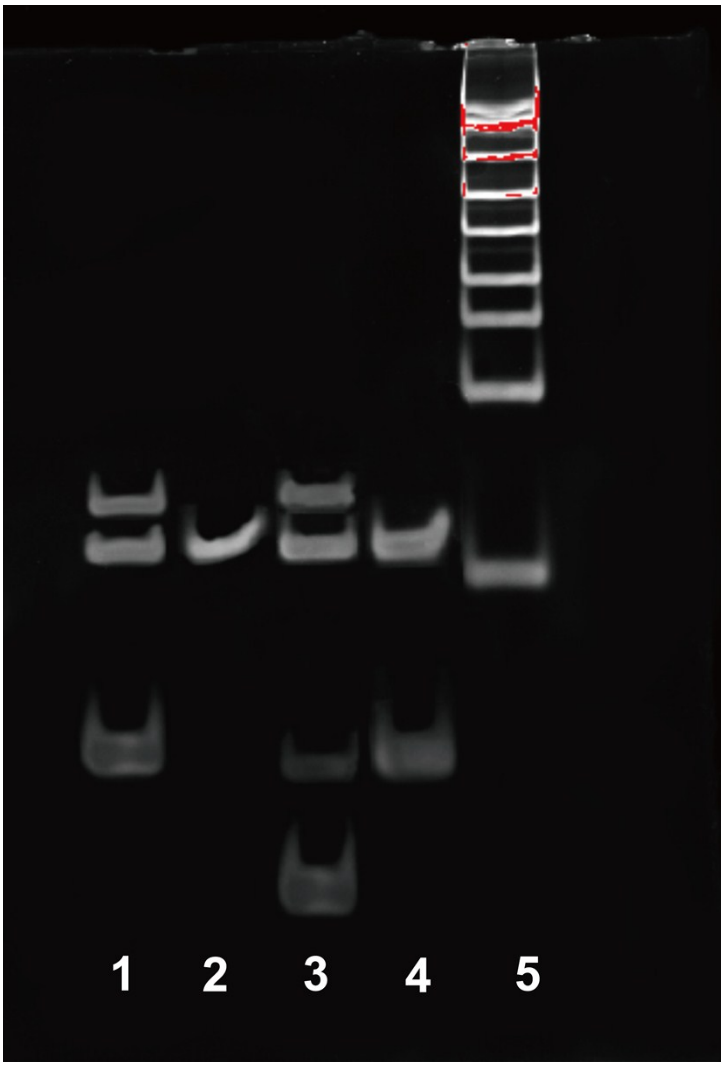

Fig. S1 native 12% polyacrylamide gel electrophoresis, lane 1: aptamer/strand a + strand b + strand c + strand d + strand e; lane 2: strand b + strand c+ strand d; lane 3: aptamer/strand a + strand b+ strand c+ strand d+ strand e + EpCAM; lane 4: strand b+ strand c + strand d + strand e; lane 5: marker.
